# Roles of the Dbl family of RhoGEFs in mechanotransduction – a review

**DOI:** 10.3389/fcell.2024.1485725

**Published:** 2024-10-16

**Authors:** Kazumasa Ohashi, Aoi Kunitomi, Shuhei Chiba, Kensaku Mizuno

**Affiliations:** Department of Molecular and Chemical Life Sciences, Laboratory of Molecular and Cellular Biology, Graduate School of Life Sciences, Tohoku University, Sendai, Miyagi, Japan

**Keywords:** RhoGEF, Dbl family, Rho, mechanotransduction, actin cytoskeleton

## Abstract

Rho guanine nucleotide exchange factors (RhoGEFs) comprise a wide range of proteins with a common domain responsible for the activation of the Rho family of small GTPases and various domains in other regions. The evolutionary divergence of RhoGEFs enables actin cytoskeletal reorganization, leading to complex cellular responses in higher organisms. In this review, we address the involvement of RhoGEFs in the mechanical stress response of mammalian cells. The cellular mechanical stress response is essential for the proper and orderly regulation of cell populations, including the maintenance of homeostasis, tissue morphogenesis, and adaptation to the mechanical environment. In particular, this review focuses on the recent findings regarding the Dbl family of RhoGEFs involved in mechanical stress responses at the cell-cell and cell-substrate adhesion sites, and their molecular mechanisms underlying actin cytoskeleton remodeling and signal transduction.

## 1 Introduction

Our bodies are constantly being subjected to a variety of mechanical forces. Cells sense these forces and trigger a range of responses that regulate various physiological functions, such as cell morphology, polarity, motility, metabolism, proliferation, differentiation, and cell death (Orr et al., 2006; Vogel and Sheetz, 2006; Jaalouk and Lammerding, 2009). Mechanical stimuli include gravity, compression, osmotic pressure, respiration, blood circulation, muscle contraction, and the force exerted by tissue and cell stiffness. The forces created by tissue and cell stiffness vary, from large forces on bones and muscles to minute forces between cells (Jaalouk and Lammerding, 2009). Cells possess diverse molecular mechanisms for sensing forces and maintaining vital activities while adapting to their mechanical environments. The molecular mechanisms underlying the sensing of mechanical stimuli, which depend on the cytoskeleton and cell adhesion and have been linked to known intracellular signaling pathways, have been elucidated (Ohashi et al., 2017; Xie et al., 2023).

Reorganization of the actin cytoskeleton is linked to almost all cellular responses, including mechanical stress responses. The Rho family of small GTPases is essential for actin cytoskeletal remodeling and acts as a molecular switch. The human genome encodes approximately 20 Rho GTPases, including RhoA, Rac1, and Cdc42, which alternate between inactive GDP-bound and active GTP-bound forms, and induce the formation of a specific actin cytoskeletal structure via physical and functional interactions with downstream effectors (dos Remedios et al., 2003; Jaffe and Hall, 2005; Heasman and Ridley, 2008; Blanchoin et al., 2014; Pollard and Goldman, 2018). It is interesting to question how such a limited number of Rho GTPases control the spatiotemporal regulation of actin cytoskeleton in response to diverse stimuli to cells. The human genome contains more than 70 Rho guanine nucleotide exchange factors (RhoGEFs), which function as activators of Rho GTPases and more than 70 Rho GTPase-activating proteins (RhoGAPs), serving as Rho GTPases inactivators. RhoGEFs and RhoGAPs are classified into protein families based on their GEF or GAP domains. They differ owing to a combination of various domains, including protein-protein interaction domains and enzymatic and membrane-binding domains. Studies have suggested that the evolutionary diversification of RhoGEFs and RhoGAPs is responsible for the specific cellular responses observed, including mechanical stress responses, which enable reorganization of the complex actin cytoskeleton with only a limited number of Rho family proteins (Tcherkezian and Lamarche-Vane, 2007; Cherfils and Zeghouf, 2013; Cook et al., 2014). While many studies have been conducted to elucidate the molecular mechanisms underlying the role of RhoGEFs in response to mechanical stresses, there is a need to represent these findings in one place that help further guide research in this area, This review focuses on the role of RhoGEFs in mechanical stress responses and describes the molecular mechanisms underlying their functions.

## 2 Molecular mechanisms underlying mechanical stimuli sensing in cells

**FIGURE 1:**
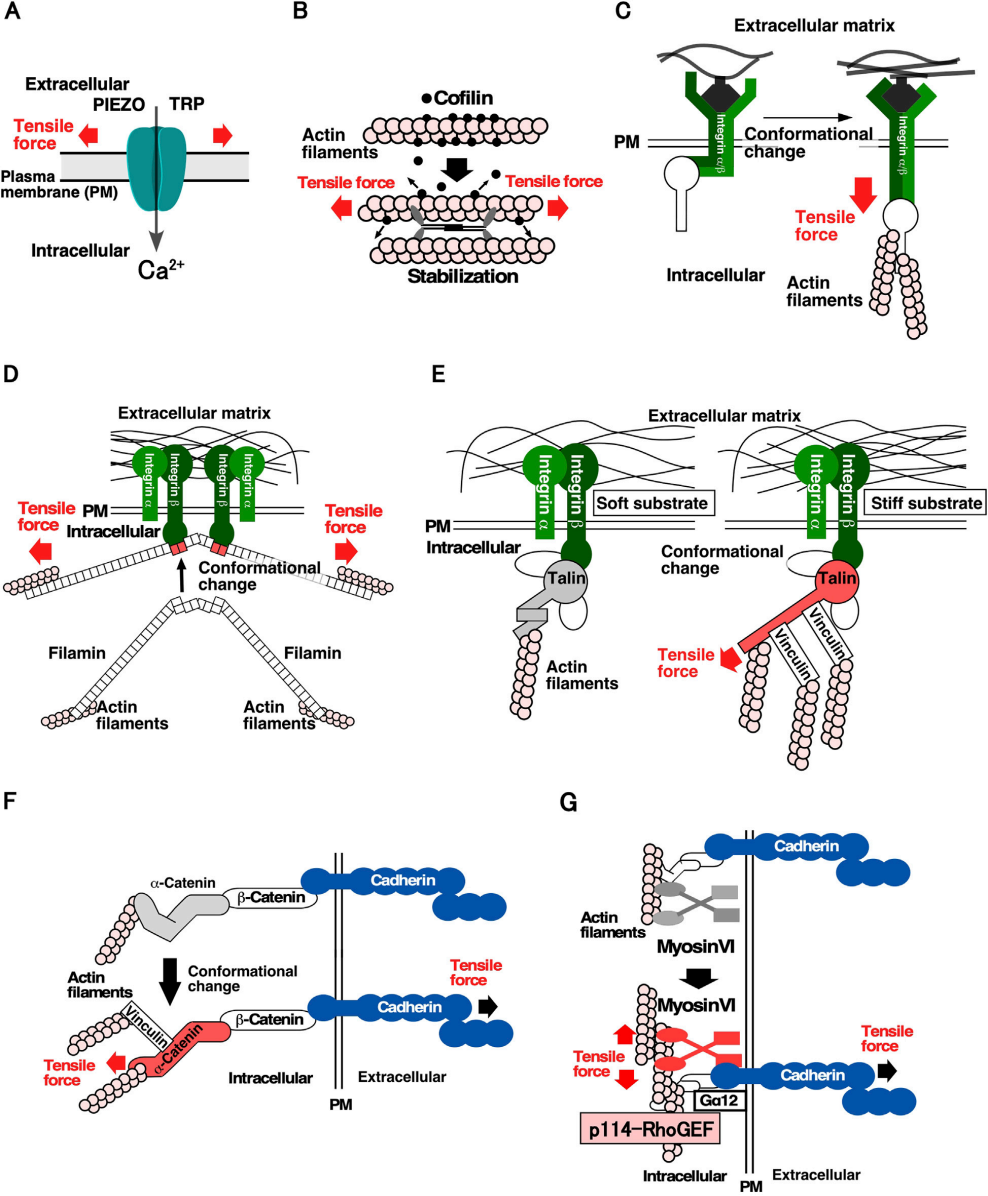
Molecular mechanisms underlying the sensing of mechanical stimuli in cells **(A)**. Mechanosensitive cation channels. The channels change their conformation in response to stretching of the plasma membrane, thereby facilitating Ca^2^⁺ permeation (Maroto et al., 2005; Coste et al., 2010). **(B)**. Actin filaments and cofilin as a tension sensor. Actin filaments subjected to tensile force are stabilized by a decrease in their affinity for cofilin, resulting in the maintenance of actin filaments in the parallel direction of the tensile force within the cell (Hayakawa et al., 2011). C, D Catch-bond effects of integrin and filamin. **(C)**. Integrin changes conformation by applying a tensile force and binds more strongly to the substrate (Kong et al., 2009). **(D)**. Filamin changes the conformation of the 20th and 21st filamin repeat domains by applying a tensile force, resulting in a high affinity for integrins (Rakshit et al., 2012; Rognoni et al., 2012). **(E)**. Talin as a sensor of substrate stiffness. Talin changes its conformation by applying a tensile force, resulting in the reinforcement of the actin structure in the focal adhesions through the binding of vinculin (del Rio et al., 2009; Elosegui-Artola et al., 2016). **(F)**. α-catenin as a tension sensor of cell-cell adhesion sites. α-catenin changes its conformation by subjecting a tensile force and strengthens the actin structure of adherens junction by binding to vinculin (Miyake et al., 2006; Yonemura et al., 2010; Huveneers and de Rooij, 2013). **(G)**. Myosin VI as a tension sensor of cell-cell adhesion sites. Force subjected to actin filaments at cell-cell adhesion sites enhances the affinity of myosin VI for E-cadherin. Subsequently, Gα12 and p114RhoGEF are recruited to cell-cell adhesion sites and RhoA is activated (Acharya et al., 2018).

For cells to detect mechanical forces, it is crucial for them to have molecules that convert mechanical forces into chemical signals. Figure 1 shows the proteins and molecular mechanisms proposed for the mechanosensing system. The Transient Receptor Potential (TRP) family and PIEZO mechanosensitive cation channels open their gates when the cell membrane is stretched, allowing an influx of Ca^2+^, increasing the Ca^2+^ concentration in local areas within the cell (Figure 1A) (Maroto et al., 2005; Coste et al., 2010). Mechanosensitive channels are speculated to be effective sensors for communicating the location, frequency, and magnitude of mechanical stimuli to cells, and contribute significantly to mechanical stress responses. Several proteins of the cytoskeleton and cell adhesion structures have been identified as mechanosensory proteins. Actin filaments linked to cell structures and adhesion sites act as mechanosensors. When the actin filaments bind to cofilin, an actin depolymerization factor, they are severed and depolymerized. However, actin filaments stretched by tensile forces are stabilized by the weakening in their binding affinity to cofilin. It has been proposed that stabilized actin filaments serve as sensors to detect the direction and magnitude of the force applied to cells (Figure 1B) (Hayakawa et al., 2011). At the cell-substrate adhesion site, integrin, an adhesion molecule, functions as a mechanosensor via the catch-bond effect, in which its binding to the substrate is strengthened by the tensile force (Figure 1C) (Kong et al., 2009). A similar catch-bond effect has been observed for filamin, an actin-bundling protein, and E-cadherin, a cell-cell adhesion molecule (Figure 1D) (Rakshit et al., 2012; Rognoni et al., 2012). Talin, which links integrin and actin filaments at focal adhesions, plays a significant role in sensing substrate stiffness. In response to tensile force, talin undergoes conformational changes that facilitate its binding to vinculin, resulting in the linking of more actin filaments via vinculin binding and the stabilization of cell adhesion to the substrate (Figure 1E) (del Rio et al., 2009; Elosegui-Artola et al., 2016). At the cell-cell adhesion sites, it was found that α-catenin, which connects E-cadherin to actin filaments at adherens junctions, undergoes a conformational change; it goes into an “open form” due to tensile forces derived from neighboring cells. Subsequently, vinculin binds to the open form of α-catenin and anchors the actin filaments, thereby strengthening cell-cell adhesion sites and generating contractile force (Figure 1F) (Miyake et al., 2006; Yonemura et al., 2010; Huveneers and de Rooij, 2013). It has been shown that cells sense the stiffness of adjacent cells through E-cadherin-based cell-cell adhesion complex and reorganize the actin cytoskeleton in a stiffness-dependent manner (Collins et al., 2017; Eftekharjoo et al., 2022; Yang et al., 2022). Moreover, myosin VI functions as a mechanosensor and promotes actomyosin formation through stimulating recruitment of Gα12 and p114RhoGEF to cell-cell adhesion sites when tension is applied to cell-cell adhesion sites (Figure 1G). This study was the first to describe the molecular link from mechanosensing to regulating RhoA activity (described below) (Acharya et al., 2018). Considering the diversity of mechanical stress responses, many other proteins are expected to function as mechanosensors.

## 3 Function of Rho family GTPases signaling in mechanical stress responses

The Rho family of small GTPases in humans consists of 20 family members, including RhoA, Rac1, and Cdc42. Previous studies have revealed their role in cellular responses to various mechanical stimuli. In particular, RhoA has been shown to play important roles in mechanical stress responses, including actin filament polymerization through pushing or pulling on the cell membrane, shear stress due to liquid flow, tensile force loading via cell-cell adhesion, and substrate stiffness (Li et al., 1999; Zhao et al., 2007; Hoon et al., 2016; Bays et al., 2017; Xie et al., 2023). However, it remains challenging to analyze the underlying molecular mechanisms of Rho-associated mechanical stress responses by manipulating the function of Rho proteins because Rho proteins are also involved in actin cytoskeletal remodeling via various intracellular signals simultaneously. Several studies on RhoGEFs have elucidated the molecular mechanisms underlying the spatiotemporal regulation of Rho pathways, and those on mechanical stress responses are gradually being revealed. Two distinct protein families serve as GEF for Rho GTPases in the human genome, comprising 69 and 11 members of the Dbl and DOCK family of proteins, respectively (Rossman et al., 2005). The Dbl family of proteins possesses a tandem structure consisting of a Dbl homology (DH) domain, which is primarily responsible for the GEF activity, and a pleckstrin homology (PH) domain, which contributes to plasma membrane localization. Many Dbl family RhoGEFs require the PH domain for GEF activity together with the DH domain. The Dbl family of proteins is diverse and contains various domains other than the DH-PH domain. In contrast, the DOCK family of proteins contains the DOCK homology regions 1 and 2 (DHR1 and 2), which exert GEF activity that is structurally distinct from the DH-PH domain (Rossman et al., 2005). Several RhoGEFs involved in mechanical stress responses have been recently identified (Table 1), most of which are RhoA-targeting RhoGEFs. Because it is easy to detect the cellular response that generates contractile force via the determination of actomyosin formation in response to forces, many RhoA-targeting RhoGEFs, which are mainly involved in the generation of contractile force via the RhoA-ROCK pathway, have been reported to be involved in mechanical responses. The following sections describe the specific RhoGEFs involved in responses to mechanical stimuli.

**TABLE 1:** RhoGEFs involved in mechanical stresses responses.

RhoGEFs	Other name	Mechanical stress	Target of RhoGTPases	References
Abr		Cyclic stretching	RhoA/Rac1/Cdc42	Abiko et al. (2015)
Alsin	ALS2	Cyclic stretching	Rac1	Abiko et al. (2015)
ARHGEF10	GEF10	Cyclic stretching	RhoA/RhoB/RhoC	Abiko et al. (2015)
Bcr	Bcr1	Cyclic stretching	RhoA/Rac1/Cdc42	Abiko et al. (2015)
GEF-H1	ARHGEF2	Cyclic stretching Tensile force on cell-substrate or cell-cell adhesion	RhoA	Birukova et al. (2010) Guilluy et al. (2011) Scott et al. (2016)
LARG	ARHGEF12	Cyclic stretching, Tensile force on cell-substrate adhesion	RhoA	Guilluy et al. (2011)
p114RhoGEF	ARHGEF18	Stretching Calyculin A treatment	RhoA/Rac1	Acharya et al. (2018)
p115RhoGEF	ARHGEF1	Tensile force on cell-cell adhesion	RhoA	Scott et al. (2016)
p190RhoGEF	ARHGEF28/RGNEF	Cyclic stretching	RhoA	Abiko et al. (2015)
PDZ-RhoGEF	ARHGEF11	Tensile force on cell-cell adhesion	RhoA	Ito et al. (2017)
PLEKHG1	ARHGEF41	Cyclic stretching	Rac1/Cdc42	Abiko et al. (2015)
P-REX2		Cyclic stretching	Rac1-3/Cdc42/RhoG/TC10	Abiko et al. (2015)
Solo	ARHGEF40/Scambio /Quo	Cyclic stretching Tensile force on cell-substrate or cell-cell adhesion	RhoA/RhoC	Abiko et al. (2015) Fujiwara et al. (2016) Isozaki et al. (2020)
α-Pix	ARHGEF6/COOL-2	Cyclic stretching, Tensile force on cell-cell adhesion	Rac1/Cdc42	Abiko et al. (2015)
β-Pix	ARHGEF7/COOL-1	Cyclic stretching Tensile force on cell-cell adhesion	Rac1/Cdc42	Abiko et al. (2015) Plutoni et al. (2016)
Vav-2		Cyclic stretching	RhoA	Peng et al. (2010)

## 4 Dbl family RhoGEFs involved in mechanical stress responses

The Dbl family of RhoGEFs activates Rho family GTPases and plays important roles in mechanical stress responses. Table 1 lists the Dbl family of RhoGEFs that have been reported to be involved in mechanical stress responses. Below, we describe the typical examples of these RhoGEFs.

### 4.1 Vav2

Based on a study into the mechanisms of hypertension-induced injury in the glomerular capillaries of the kidney, it was found that Vav2 is involved in cyclic stretching-induced RhoA activation (Peng et al., 2010). RhoA is activated in mesangial cells, the supporting cells of glomerular capillaries, by cyclic stretching that mimics changes in blood pressure. Therefore, a RhoGEF that causes the activation of RhoA was investigated. Furthermore, because RhoA activation by cyclic stretching requires Src, a non-receptor-type protein tyrosine kinase, the Vav family of RhoGEFs, activated by Src-mediated phosphorylation, was investigated (Barfod et al., 2005). These studies demonstrated that Src phosphorylates Tyr-172 of Vav2, thereby inducing RhoA activation. This was the first report to identify a RhoGEF involved in mechanotransduction (Peng et al., 2010).

### 4.2 GEF-H1 (also known as ARHGEF2)

Microtubule networks present in the vascular endothelial cells of the pulmonary artery play a crucial role in maintaining the barrier function of the cell layers against cyclic stretching (Kaunas et al., 2005). GEF-H1, a microtubule-associated RhoGEF, is activated by its dissociation from microtubules (Krendel et al., 2002). Furthermore, other studies have revealed that GEF-H1 is required for cell orientation and RhoA activation in response to cyclic stretching (Birukova et al., 2010). Subsequently, GEF-H1 has been shown to be involved in integrin-mediated mechanical stress responses. To identify RhoGEFs necessary for this mechanical stress response, Guilluy et al. utilized a model of mechanical stress response, whereby fibronectin-coated magnetic beads adhered to fibroblasts were stretched by magnetic force, causing stiffening around the bead-attached site. Using this approach, they identified GEF-H1 and Leukemia-associated RhoGEF (LARG; also named as ARHGEF12) as RhoGEFs involved in mechanical stress response (Guilluy et al., 2011). They showed that GEF-H1 activation in response to a tensile force stimulus requires focal adhesion kinase (FAK) and mitogen-activated protein kinase kinase (MEK), using their inhibitors. GEF-H1 is involved in RhoA activation in response to tension applied at cell-cell adhesion sites (Guilluy et al., 2011). Magnetic beads coated with antibodies specific to JAM-A, an immunoglobulin superfamily intercellular adhesion protein, were attached to cells and pulled by magnetic force to mimic JAM-A-dependent tension loading at cell-cell adhesion sites, resulting in RhoA activation. Using this assay as a model, GEF-H1 and p115RhoGEF (also named as ARHGEF1) were shown to be involved in RhoA activation in response to tensile force at cell-cell adhesion sites (Scott et al., 2016).

### 4.3 RGS-RhoGEF family

LARG, p115RhoGEF, and PDZ-RhoGEF (PRG, also named as ARHGEF11) are the members of a subfamily of the Dbl family that have the regulator of G protein signaling (RGS) domain at the N-terminus (Rossman et al., 2005; Siehler, 2009). The RGS domain binds to the α-subunit of trimeric G proteins, Gα12/Gα13, which are activated downstream of G protein-coupled receptors (GPCRs). Upon binding of the α-subunit of Gα12/Gα13 to the RGS domain of these RhoGEFs, these RhoGEFs are activated, leading to the activation of RhoA and the suppression of alpha subunit activity (Hart et al., 1998). These three RhoGEFs are involved in mechanical stress response. As described above, LARG activates RhoA in response to the tensile force applied to cells through integrin, and p115RhoGEF is required for the activation of RhoA in response to the tensile force applied to cell-cell adhesion through JAM-A (Guilluy et al., 2011; Scott et al., 2016). PRG localizes to the intercellular adhesion sites upon activation of RhoA via microtubule depolymerization, suggesting that PRG is involved in the mechanical regulation of intercellular adhesion structures (Ito et al., 2017). It has also been reported that Gα12/Gα13 is required for RhoA activation upon the stretching of cardiomyocytes and other cells, as well as the signal from GPCR, suggesting that Gα12/Gα13 can receive signals from upstream mechanosensors to activate RGS-RhoGEFs (Nishida et al., 2008). Additionally, it was found that PRG cooperates with Solo, a RhoGEF involved in force response and response to substrate stiffness, as described below (Kunitomi et al., 2024).

### 4.4 p114RhoGEF (also named as ARHGEF18)

Yap and colleagues established an experimental model for applying tensile force to the cell-cell adhesion sites of a monolayer of male human colorectal Caco-2 cells by activating actomyosin using calyculin A, an inhibitor of myosin light chain phosphatase, and stretching the cell layer (Acharya et al., 2018). p114RhoGEF regulates RhoA activity at the cell-cell adhesion sites of epithelial cells, and is involved in the generation of contractile forces in the actomyosin ring surrounding the apical side of the cell-cell adhesion sites in the layered structure of the epithelial cell population (Niu et al., 2003; Nakajima and Tanoue, 2011; Terry et al., 2011). Therefore, the function of p114RhoGEF was investigated using this model, which revealed that p114RhoGEF accumulates at cell-cell junctions in response to the tensile forces and activates RhoA. In addition, since p114RhoGEF was reported to be one of the RhoGEFs that functions downstream of trimeric G proteins, its correlation with Gα12 was investigated, revealing that Gα12 also accumulates at cell-cell junctions upon tensile force application, and the force-induced translocation of p114RhoGEF at the cell-cell adhesion sites depends on Gα12 (Acharya et al., 2018). Furthermore, the study showed that the motor protein myosin VI is required for the tension-dependent accumulation of Gα12 at the intercellular adhesion sites, and that the binding of myosin VI to E-cadherin specifies the localization of these proteins at these sites (Acharya et al., 2018). It has been proposed that myosin VI functions as a tension sensor by altering its ATPase activity when bound to actin filaments and subjected to a force (Chuan et al., 2011). This report showcases a significant discovery as it was the first to demonstrate the signaling pathway from a mechanosensor protein to RhoGEF.

### 4.5 Solo (also named as ARHGEF40)

Solo is a GEF for RhoA and RhoC and was discovered in a yeast two-hybrid screen as a RhoGEF that binds to Rac3 (Curtis et al., 2004). Moreover, Quattro, an ortholog of Solo is necessary for the convergent extension of mesodermal cells during gastrulation in the early development of zebrafish (Daggett et al., 2004). Following a knockdown screen of the Dbl family of RhoGEFs involved in mechanical stress responses using a model in which vascular endothelial cells were oriented in a direction perpendicular to the cyclic stretching axis, Solo and 10 other RhoGEFs, including LARG and GEF-H1, were identified (Table 1) (Abiko et al., 2015). Furthermore, proteomic analysis revealed that Solo binds to Keratin8/Keratin18 (K8/K18) filaments, cytoplasmic intermediate filaments specifically expressed in simple epithelia, and this binding is required for tensile force-induced RhoA activation (Fujiwara et al., 2016). This was the first study revealing that RhoGEF functions in the mechanical stress response by coordinating the actin cytoskeleton and intermediate filaments. Furthermore, Solo knockdown accelerates the collective migration of MDCK cells (Isozaki et al., 2020). Depletion of LARG, p115RhoGEF, K8/K18, and several desmosome proteins similarly increase the velocity of collective cell migration, suggesting that these RhoGEFs, including Solo, and desmosome structures contribute to the generation of tensile forces and force equilibration at cell-cell adhesion sites (Medlin et al., 2010; Kher et al., 2014). Proteomic studies have indicated that Solo interacts with PRG (Muller et al., 2020). Our recent research has further emphasized the functional significance of the interaction of these proteins. Specifically, we found that PRG colocalized with Solo in the basal area of the cell, where a contractile force was generated. Moreover, our findings revealed that Solo directly binds to PRG and activates its GEF activity for RhoA (Kunitomi et al., 2024). This is the first study to demonstrate that the novel RhoGEF cascade from Solo to PRG is activated in response to substrate stiffness.

### 4.6 Pleckstrin homology domain containing RhoGEF G4B (PLEKHG4B)

PLEKHG4B is a RhoGEF that belongs to the Solo subfamily of the Dbl family and shares sequence and domain structural similarities with Solo. However, unlike Solo, PLEKHG4B targets another member of the Rho family, Cdc42 (Gupta et al., 2013; Cook et al., 2014). A comprehensive analysis of PLEKHG4B interactors elucidated its interaction with LARG and PRG, and demonstrated that, in contrast to Solo, PLEKHG4B inhibits the GEF activity for RhoA (Muller et al., 2020). We found that PLEKHG4B is required for the maturation of epithelial cell-cell adhesion via binding to annexin A2 (Ninomiya et al., 2021). Furthermore, we showed that PLEKHG4B localizes to the basal sites of cell-cell adhesion in a Ca^2+^ influx-dependent manner through mechanosensitive cation channels (Ninomiya et al., 2024). PLEKHG4B is suggested to be a RhoGEF that functions specifically downstream of mechanosensitive channels and Ca^2+^ influx at cell-cell adhesion sites.

### 4.7 β-Pix (also named as ARHGEF7)

P-cadherin contributes to the direction and generation of tensile forces at cell-substrate and cell-cell adhesion sites in collectively migrating cancer cells, indicating that P-cadherin is involved in mechanical stress responses in migrating cell population (Plutoni et al., 2016). β-Pix has been identified as a binding protein for P-cadherin and is thought to be required for P-cadherin-dependent force generation during collective migration. It is also required for P-cadherin-dependent activation of Cdc42 during collective cell migration (Plutoni et al., 2016). This report demonstrates that a RhoGEF for Cdc42, although indirectly, functions in response to tensile forces during cell-cell adhesion.

## 5 Conclusion and perspective

The molecular mechanisms involving various molecules that regulate the mechanical stress response are currently being elucidated. However, only a few molecules and mechanisms that function as sensors have been identified. Since RhoGEF proteins, along with RhoGAPs, are a diverse group of molecules that regulate actin cytoskeleton remodeling, it is speculated that there are several GEFs and GAPs in which mechanical forces alter the activity of the GEFs and GAPs. By accurately predicting the three-dimensional structures of molecules and their mechanical properties, it would be possible to predict the proteins and their motifs that function as mechanosensors. Further research investigating the mechanisms by which these sensor proteins regulate Rho proteins is expected to reveal the comprehensive molecular mechanisms of mechanical stress responses. It has been suggested that proteins involved in mechanical stress responses are associated with the initiation and malignant transformation of cancer and other diseases, owing to defects in metabolic control and tissue barrier function (Xie et al., 2023). Therefore, studying the molecular mechanisms of mechanical stress responses is expected to become even more important in the future to elucidate the causes of diseases and provide effective therapeutic treatments.
